# Upregulation of Claudin-4, CAIX and GLUT-1 in distant breast cancer metastases

**DOI:** 10.1186/1471-2407-14-864

**Published:** 2014-11-22

**Authors:** Laura S Jiwa, Paul J van Diest, Laurien D Hoefnagel, Jelle Wesseling, Pieter Wesseling, Dutch Distant Breast Cancer Metastases Consortium, Cathy B Moelans

**Affiliations:** Department of Pathology, University Medical Center Utrecht, Heidelberglaan 100, PO Box 85500, Utrecht, 3508GA The Netherlands

**Keywords:** Claudin-4, CAIX, GLUT-1, Receptor conversion, Breast cancer

## Abstract

**Background:**

Several studies have shown that the immunophenotype of distant breast cancer metastases may differ significantly from that of the primary tumor, especially with regard to differences in the level of hormone receptor protein expression, a process known as receptor conversion. This study aimed to compare expression levels of several membrane proteins between primary breast tumors and their corresponding distant metastases in view of their potential applicability for molecular imaging and drug targeting.

**Methods:**

Expression of Claudin-4, EGFR, CAIX, GLUT-1 and IGF1R was assessed by immunohistochemistry on tissue microarrays composed of 97 paired primary breast tumors and their distant (non-bone) metastases.

**Results:**

In both the primary cancers and the metastases, Claudin-4 was most frequently expressed, followed by GLUT-1, CAIX and EGFR.

From primary breast cancers to their distant metastases there was positive to negative conversion, e.g. protein expression in the primary tumor with no expression in its paired metastasis, in 6%, 19%, 12%, 38%, and 0% for Claudin-4 (n.s), GLUT-1 (n.s), CAIX (n.s), EGFR (n.s) and IGF1R (n.s) respectively. Negative to positive conversion was seen in 65%, 47%, 43%, 9% and 0% of cases for Claudin-4 (p = 0.049), GLUT-1 (p = 0.024), CAIX (p = 0.002), EGFR (n.s.) and IGF1R (n.s.) respectively. Negative to positive conversion of Claudin-4 in the metastasis was significantly associated with tumor size (p = 0.015), negative to positive conversion of EGFR with negative PR status (p = 0.046) and high MAI (p = 0.047) and GLUT-1 negative to positive conversion with (neo)adjuvant chemotherapy (p = 0.039) and time to metastasis formation (p = 0.034). CAIX and GLUT-1 expression in the primary tumor were significantly associated with high MAI (p = 0.008 and p = 0.038 respectively).

**Conclusion:**

Claudin-4 is frequently expressed in primary breast cancers but especially in their metastases and is thereby an attractive membrane bound molecular imaging and drug target. Conversion in expression of the studied proteins from the primary tumor to metastases was fairly frequent, except for IGF1R, implying that the expression status of metastases cannot always be reliably predicted from the primary tumor, thereby necessitating biopsy for reliable assessment.

## Background

Breast cancer is the most prevalent cancer among women worldwide. The lifetime risk to develop breast cancer in The Netherlands is 1 in 8 for women and 1 in 1,000 for men [1, 2]. Due to the increased life expectancy and the changing age distribution in The Netherlands, the incidence of breast cancer is increasing every year. As a result of early diagnosis and improved local and systemic treatment, the five-year survival rate of breast cancer has increased over the last 20 years. Still, breast cancer is the leading cause of cancer mortality in women worldwide [3], in which progression of metastases plays a key role.

To improve early detection of distant metastases and their molecular characterization, recent research has focused on molecular imaging techniques such as positron emission tomography (PET), single photon emission computed tomography (SPECT) and optical fluorescence imaging [4] by targeting specific (membrane) proteins. In addition, drugs targeting such proteins are widely under development.

However, several studies have shown that the immunophenotype of distant breast cancer metastases may differ significantly from that of the primary tumor, especially with regard to differences in the level of hormone and human epidermal growth factor receptor 2 (HER2) protein expression, a process known as receptor conversion [5, 6]. This phenomenon may be clinically relevant as the consequence of this receptor conversion may be that some patients with distant metastases are withheld adequate therapy or receive expensive unnecessary or inadequate therapy with possible side-effects. In addition, it has been shown that conversion of the estrogen receptor (ER) from positive in the primary breast tumor to negative in its metastasis counterpart is associated with a worse prognosis [7].

Therefore, it cannot just be assumed that molecular imaging and drug targets that are present in the primary tumor are retained in their distant breast cancer metastases, and the other way around. This may lead to false negative molecular imaging results and may deny patients proper personalized cancer treatment of metastases.

To this end, we set out to compare expression levels of five tumor specific membrane bound candidate imaging [8–11] and drug targets (Claudin-4, the Epidermal Growth Factor Receptor (EGFR), Carbonic Anhydrase IX (CAIX), the Glucose Transporter 1 (GLUT-1) and the Insulin Growth Factor Receptor 1 (IGF1R) in primary breast cancers and their distant metastases, and hypothesised how this would impact molecular imaging and targeted therapy.

## Methods

### Patient material

From a previously described group of 254 patients with paired primary breast cancer and (non-bone) metastases, 97 pairs eligible for manufacturing tissue microarrays (TMAs) were selected, based on availability of material (e.g. biopsies with too little tissue were excluded). Representative areas containing primary or metastatic carcinoma (lung, brain, liver, skin, ovary, cervix, uterus, endometrium, stomach, ileum, colon, cecum, appendix, subcutis, omentum, pleura, and peritoneum) were marked on H&E stained glass slides and used as a guide for sampling of three cores from the paraffin blocks with an automatic tissue puncher and arrayer (TMA Grand Master, 3D Histech, Sysmex Belgium N.V). Use of anonymous or coded left over material for scientific purposes is part of the standard treatment contract with patients and therefore ethics approval and informed consent procedure was not required according to Dutch legislation (Medical Research Involving Human Subjects Act, http://www.ccmo.nl and http://www.ccmo.nl/en/).

### Immunohistochemistry

Four-μm-thick sections were serially cut from the TMAs blocks, mounted on pre-coated slides and dried for at least 10 minutes at 56°C. Subsequently, sections were deparaffinized and rehydrated by a series of xylene and ethanol. Endogenous peroxidase activity was blocked by 15 minutes incubation in 1,5% (Claudin-4, EGFR, CAIX and GLUT-1) or 3% (IGF1R) H_2_O_2_ in phosphate buffer. Antigen retrieval for EGFR was performed by Proteinase K (Dako, Glostrup, Denmark) for 5 minutes and henceforth incubation with a protein block (Novolink kit; Novocastra, RE7102) for 5 minutes. For Claudin-4, CAIX and GLUT-1 antigen retrieval was performed by boiling in citrate buffer pH 6.0 for 20 minutes, followed by a cooling down period of 30 minutes. For IGF1R, slides were boiled in EDTA pH 9.0 for 20 minutes. Next, the primary antibodies to EGFR (Zymed, 28–8763, clone 31G7, 1:50), Claudin-4 (Invitrogen 32–9400, clone 3E2C1, 1:100), CAIX (Abcam, Ab15086, 1:1000), GLUT-1 (Dako, A3536, 1:200) and IGF1R (Novus Biologicals Cambridge, NB110-87052, 1:400) were incubated overnight (EGFR) or for 60 minutes at room temperature (Claudin-4, CAIX, GLUT-1 and IGF1R). Hereafter, sections were incubated with a Post Primary Block from the Novolink kit (Novocastra, RE7111) for EGFR, or with the secondary antibody Brightvision poly-HRP anti-mouse, rabbit, rat (Immunologic, Duiven, The Netherlands, DPVO500HRP), for 30 minutes in case of Claudin-4, CAIX, GLUT-1 and IGF1R. Next, sections were incubated with a Novolink polymer (Novocastra, RE7112) for 30 minutes and with the Novolink DAB kit for 5 minutes (EGFR), or directly incubated with DAB substrate for 10 minutes (Claudin-4, CAIX, GLUT-1 and IGF1R). Subsequently sections were counterstained with haematoxylin, dehydrated in graded ethanol and xylene and coverslipped.

Scoring was done by consensus of two observers including an experienced pathologist (PvD), who were blinded to patient characteristics and results of other stainings. Included positive controls comprised normal breast tissue for Claudin-4, tissue harvested from mice injected with human tumor cells expressing CAIX for CAIX, placenta tissue for GLUT-1 and tissue from breast cancer for EGFR and IGFR. Negative controls were obtained by omission of the primary antibodies.

A case was considered positive if at least one of the three cores per sample showed any membrane staining. Cases with cytoplasmatic staining in either the primary tumor or paired metastases were left out of the analysis.

Conversion from negative in the primary tumor to positive in the metastases or vice versa was noted.

### Statistics

Statistical analysis was performed using IBM SPSS Statistics 20. Paired analysis of categorical variables was performed using McNemar’s test. Unpaired associations between categorical variables were examined using the Pearson’s Chi square test or the Fisher’s Exact test when necessary.

ER, PR, HER2, mitotic activity index (MAI) and age were dichotomized using traditional cut-off points: 10% for ER and PR, 3+ for HER2, 13 for MAI and 50 for age. Two-sided p-values <0.05 were considered to be statistically significant.

## Results

### Expression of molecular imaging and drug targets

**Figure 1:**
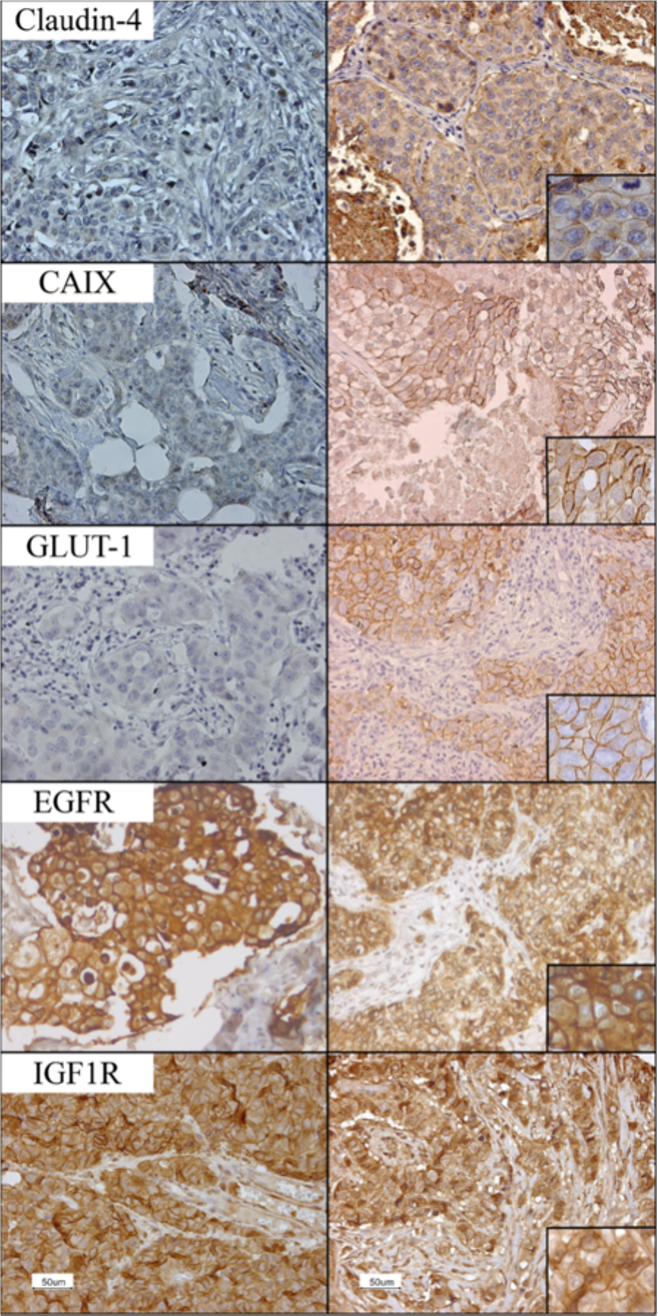
**Representative IHC staining in primary breast tumors (left; no membrane staining for Claudin-4, CAIX and GLUT-1 (significant negative to positive conversion), positive membrane staining for EGFR and IGF1R (negative to positive conversion n.s.) and metastases (right; positive membrane staining).** Magnification: 20× (inlet 40×).

Table 1 shows the expression frequencies of the different targets in primary breast tumors and distant metastases. In both the primary cancers and the metastases, Claudin-4 was most frequently expressed, followed by GLUT-1, CAIX, EGFR and IGF1R. Figure 1 illustrates representative examples of Claudin-4, CAIX and GLUT-1 expression in primary breast tumors and distant metastases.

**Table 1:** **Expression frequencies of Claudin-4, EGFR, CAIX, GLUT-1 and IGF1R in primary breast tumors and distant metastases**

	Primary cancers	Metastases
Claudin-4	68/88 (77.3%)	77/88 (87.5%)
EGFR	8/77 (10.4%)	11/77 (14.3%)
CAIX	17/52 (32.7%)	30/52 (57.7%)
GLUT-1	43/88 (48.9%)	56/88 (63.6%)
IGF1R	1/96 (1.0%)	1/96 (1.0%)

Table 2 shows the conversion rates of Claudin-4, EGFR, CAIX, GLUT-1 and IGF1R.

**Table 2:** **Conversion rates for Claudin-4, EGFR, CAIX, GLUT-1 and IGF1R**

	Conversion			
	None	Positive to negative	Negative to positive	p-value
Claudin-4	71	4 (5.8%)	13 (65%)	p = 0.049
EGFR	68	3 (37.5%)	6 (8.7%)	p = 0.508
CAIX	35	2 (11.8%)	15 (42.9%)	p = 0.002
GLUT-1	59	8 (18.6%)	21 (46.7%)	p = 0.024
IGF1R	95	0/95 (0%)	0 (0%)	P = 1.000

Membrane expression of Claudin-4, CAIX and GLUT-1 was significantly more frequent in the metastases (p = 0.049, p = 0.002 and p = 0.024 respectively), while the difference for EGFR and IGF1R was not significant (p = 0.508 and p = 1.000 respectively). 13/20 (65%) primary tumors without membrane staining for Claudin-4 had positive metastases. For EGFR, CAIX, GLUT-1 and IGF1R, the negative to positive conversion rates were 6/69 (8.7%), 15/35 (42.9%), 21/45 (46.7%), and 0/95 (0%) respectively. Positive to negative conversion rates were lower: 4/68 (5.8%), 3/8 (37.5%), 2/17 (11.8%), 8/43 (18.6%) and 0/95 (0%) for Claudin-4, EGFR, CAIX, GLUT-1 and IGF1R respectively.

### Association with clinicopathological features

Claudin-4 negative to positive conversion in the metastases was significantly associated with tumor size (p = 0.015; 11/14 versus 31/72), whereas negative to positive conversion of EGFR was associated with negative PR status (p = 0.046; 8/8 versus 46/75) and high MAI (p = 0.047; 8/8 versus 47/75). GLUT-1 negative to positive conversion was significantly associated with (neo)adjuvant chemotherapy (p = 0.039; 13/18 versus 20/46 ) and time to metastasis formation (p = 0.034; 29,8 versus 40,1 months). In addition, CAIX and GLUT-1 expression in the primary tumor was significantly associated with high MAI (p = 0.008; 20/23 versus 40/71 and p = 0.038; 34/46 versus 25/47).

No associations were found between either of these biomarkers, whether in the primary tumor or the metastasis individually or with conversion, and ER status, HER2 status, lymph node status and age. The numbers of available samples of different metastatic locations were too small for statistical analysis.

## Discussion

The aim of this study was to explore conversion of expression of a set of membrane bound molecular imaging and drug targets in distant (non-bone) breast cancer metastases. Such conversion was previously described for ER, PR and HER2 receptors [5], but was not studied before for other candidate molecular imaging and drug targets. In this manuscript we therefore analysed the membrane-bound candidate protein markers Claudin-4, EGFR, CAIX, GLUT-1 and IGF1R.

In both the primary tumor and the metastases, Claudin-4 was most frequently expressed, followed by GLUT-1, CAIX, EGFR and IGF1R. Expression of Claudin-4, CAIX and GLUT-1 was significantly more frequent in distant metastases compared to their primary tumors (negative to positive conversion). This illustrates that these proteins are potential molecular imaging and drug targets for distant breast cancer metastases, even more than for their primary tumors [8].

Claudin-4 is a membrane bound marker and is upregulated in many tumors, including breast cancer and its metastases [12, 13]. Claudins represent the structural backbones of tight junctions [14] and have distinctive functions, such as ensuring cell adhesion. Loss of cell adhesion and communication constitutes one hallmark of malignancy and may reflect a more aggressive phenotype [15]. For example, loss of Claudin-4 from the cell surface has been shown to coincide with disintegration of fibrils at tight junctions and increased junctional permeability [16]. In addition, Claudin-4 expression in undifferentiated or poorly differentiated carcinomas [17] is lower than in well-differentiated counterparts. On the other hand, while loss of Claudin-4 and other members of the Claudin family may facilitate invasion by increasing cell mobility, re-expression of Claudin-4 may confer survival advantages within host tissue, potentially by promoting cohesion in established metastases [18]. As these events are also related to tumor size [19], this might explain the finding of the significant association of Claudin-4 and larger tumor size.

EGFR belongs to the epidermal growth factor receptor subfamily of tyrosine kinase receptors. Its upregulation has been associated with epithelial proliferation, low response to hormone therapy and a poor prognosis in patients with node-negative breast cancer [20, 21]. In addition, EGFR is linked to higher mitotic rate and a shorter relapse free interval and survival [22] and triple negative and basal-like breast tumors [23, 24]. Negative to positive conversion for EGFR occurred especially in high MAI and PR negative cases.

CAIX and GLUT-1 are both hypoxia-upregulated proteins [25]. CAIX catalyzes the conversion of CO2 to bicarbonate and proton and is therefore involved in pH homeostasis, which is often deregulated as a result of hypoxia [26]. Besides that, CAIX is also involved in cell-adhesion, growth and tumor cell survival [27]. GLUT-1 is a glucose transporter that shows elevation of expression during hypoxic and acidotic conditions [28]. The negative to positive conversion of CAIX and GLUT-1 in distant metastases may be related to their key role in pH homeostasis and neo-angiogenesis, as downstream targets of hypoxia inducible factor 1α (HIF-1α) [29, 30], the key regulator of the hypoxia response which can be maintained by IGF1R [31–33]. A preferential selection of CAIX and GLUT-1 positive subclones in the primary tumor upon metastasis or adaptation to the local hypoxic environment may explain their negative to positive conversion in distant breast cancer metastases and might also explain the fact that GLUT-1 was positively associated with time to metastasis formation, as these processes might take time to develop and occur. This phenomenon could equally explain why primary tumors expressing CAIX or GLUT-1 show a higher MAI, as these tumors have a favourable proliferation profile.

Negative to positive conversion of GLUT-1 in the metastasis was seen preferentially in cases that received (neo)adjuvant chemotherapy which may be related to the resulting genetic drift [34] and treatment-related clone selection [35, 36].

CAIX and GLUT-1 expression in the primary tumor were associated with a high MAI, which can be explained.

Preanalytical (e.g. fixation and processing) and analytical (e.g. staining and scoring of IHC) variability have to be taken into account as possible confounding factors in this study [37–39]. In addition, intratumoral heterogeneity may lead to a false-positive or false-negative result in a small biopsy sample [40], as is the case in tissue microarrays. Nonetheless, the use of tissue microarrays is accepted in diagnostic and prognostic biomarker studies [41]. A general approach is that a cohort or database with larger sample numbers allows smaller core diameters [42] and as a consequence most studies use 2–3 cores of 0.6 mm per sample [43], as was the case for our study. Since EGFR overexpression is fairly rare in breast cancer [20], the sample size in the present study might have been insufficient to render significant results for this specific biomarker.

## Conclusion

Claudin-4 is frequently expressed in primary breast cancers but especially in their metastases and is thereby an attractive membrane bound molecular imaging and drug target. Conversion in expression of the studied proteins from the primary tumor to metastases was fairly frequent, except for IGF1R, implying that the expression status of metastases cannot well be predicted from the primary tumor, probably necessitating biopsy for reliable assessment.
